# Supplementary figures and images for: High expression of stromal signatures correlated with macrophage infiltration, angiogenesis and poor prognosis in glioma microenvironment
Source: PeerJ. 2020 May 20;8:e9038. doi: 10.7717/peerj.9038 (PMC7245335; doi:10.7717/peerj.9038)

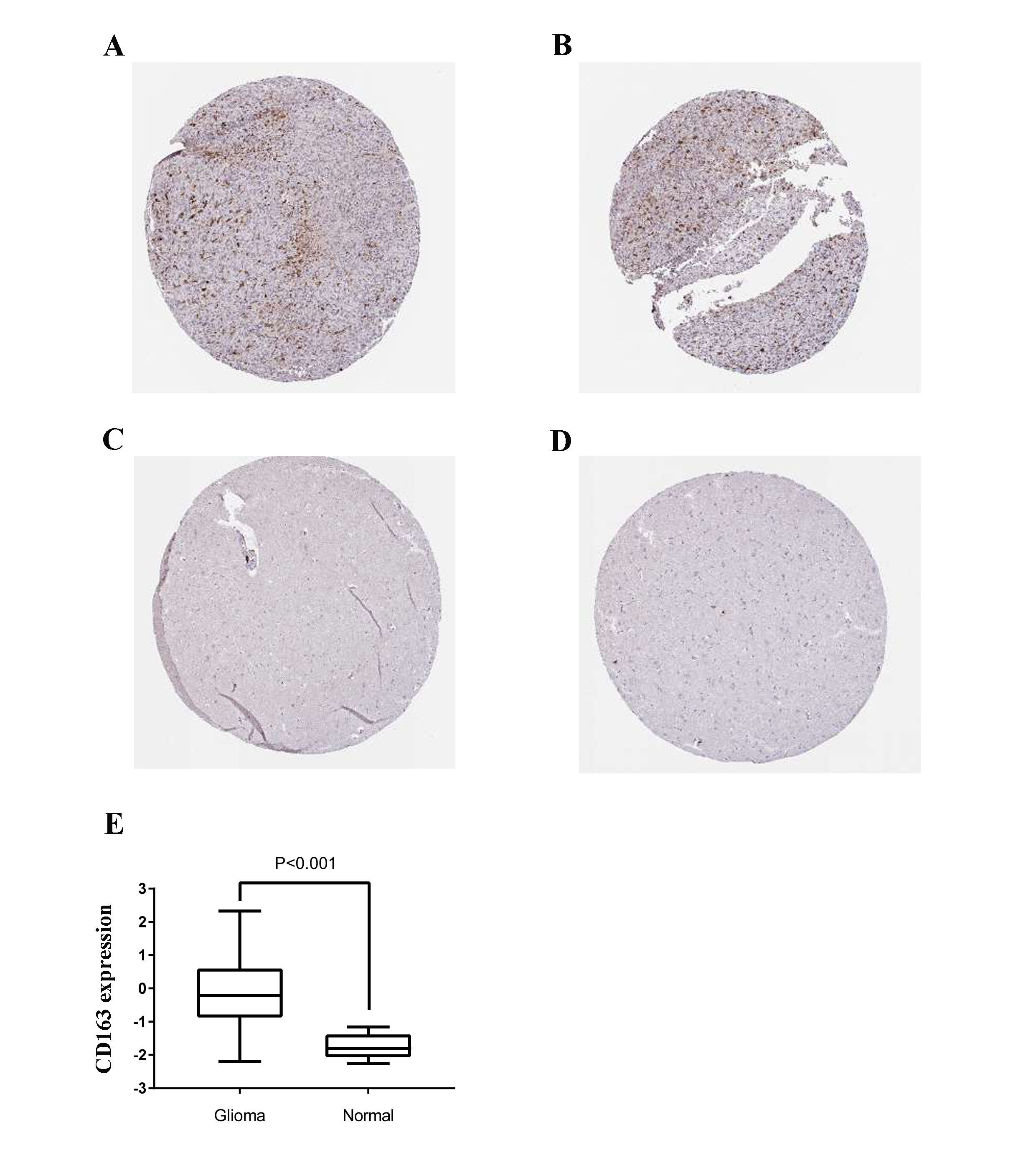

Supplement: Supplemental Information 1 — (A and B) The immunohistochemistry of CD163 in glioma patients. (C and D) The immunohistochemistry of CD163 in normal brain tissue. (E) Box plot shows the different expression of CD163 protein expression in glioma and normal tissue via CPTAC. [file peerj-08-9038-s001.png]

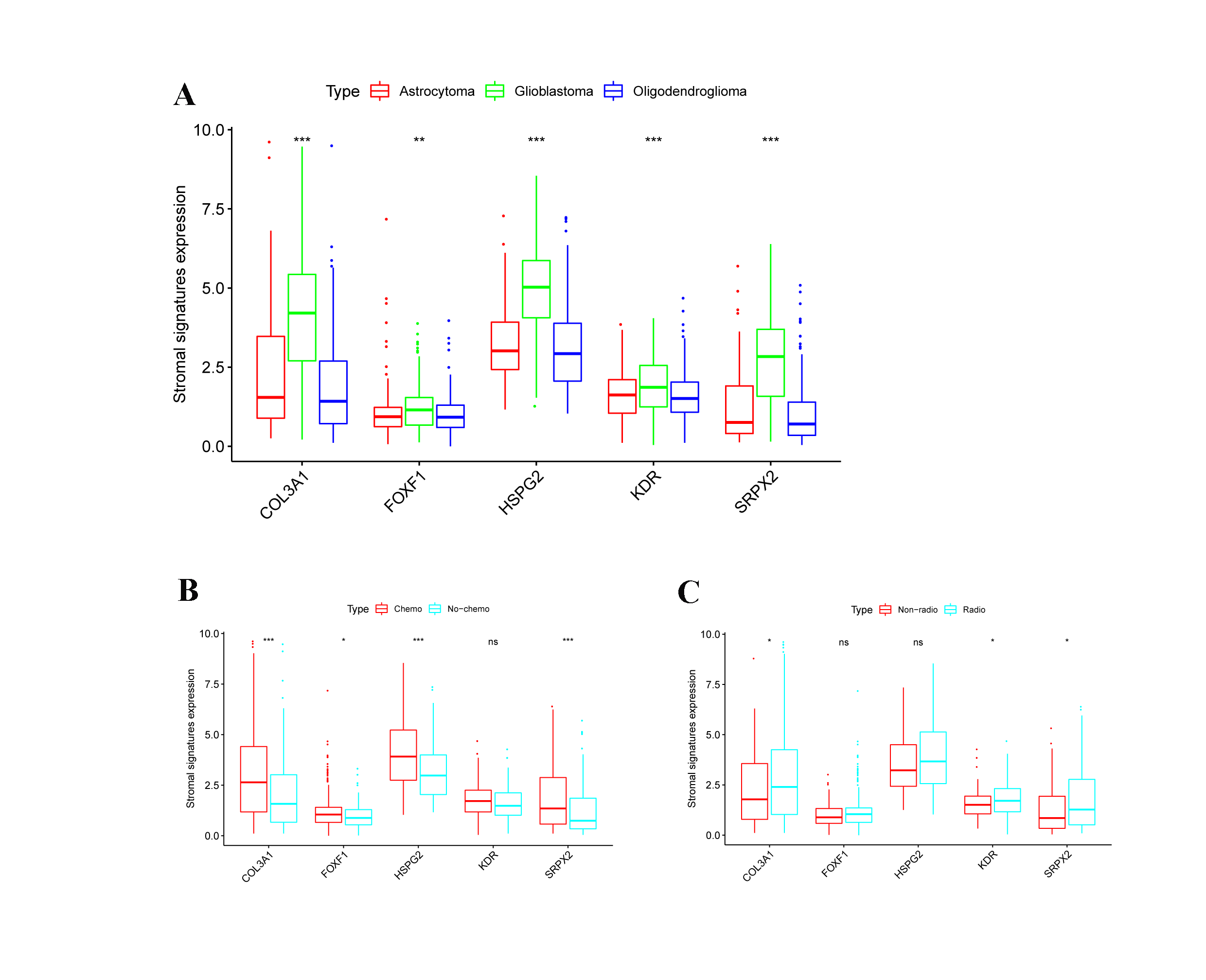

Supplement: Supplemental Information 2 — (A) Box plot shows 5 stromal signatures were correlated with histology. (B and C) Box plot shows 5 stromal signatures were correlated with radio and chemo treatment. [file peerj-08-9038-s002.png]

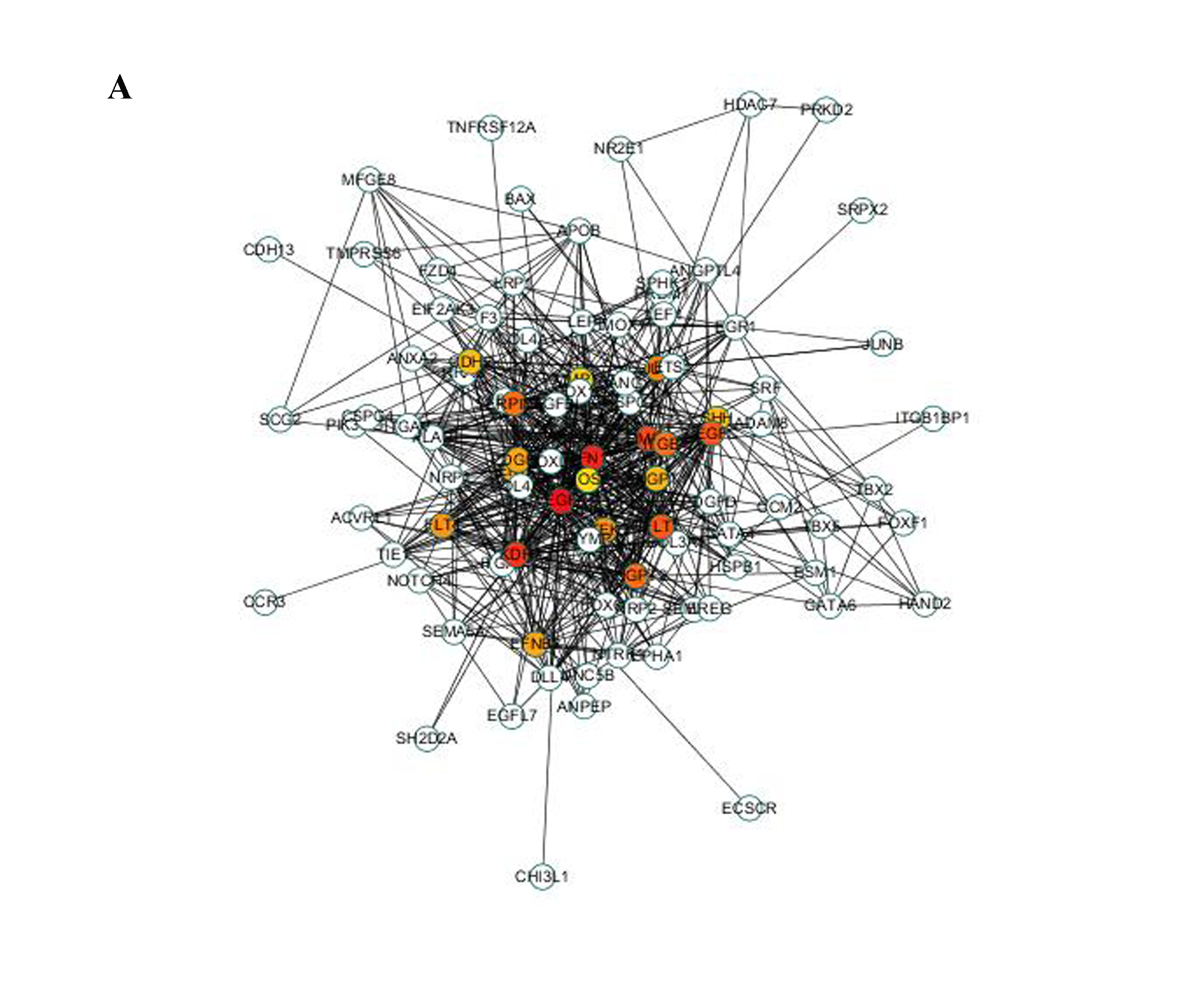

Supplement: Supplemental Information 3 [file peerj-08-9038-s003.png]
